# Exploring the Application of Intersectionality as a Path toward Equity in Perinatal Health: A Scoping Review

**DOI:** 10.3390/ijerph20010685

**Published:** 2022-12-30

**Authors:** Tuyet-Mai H. Hoang, Ainslee Wong

**Affiliations:** 1 School of Social Work, University of Illinois at Urbana-Champaign, Urbana, IL 61801, USA; 2 Department of Psychology, University of Illinois at Urbana-Champaign, Champaign, IL 61820, USA

**Keywords:** intersectionality, perinatal health, health disparities, applied health science

## Abstract

Objective: To conduct a scoping review to determine how past studies have applied the theory of intersectionality, a critical feminist research paradigm, to understand the physical health and mental health outcomes of perinatal people as a step toward addressing maternal health disparities and injustice. The study includes a review of existing research on maternal physical and mental health outcomes, presents the strengths and limitations of existing studies, and provides recommendations on best practices in applying intersectionality in research to address systemic issues and improve outcomes for the perinatal population. Methods: We conducted an extensive literature search across four search engines, yielding 28 publications using the intersectionality framework that focused on the outcomes of perinatal people, with a total sample of 9,856,042 participants. We examined how these studies applied intersectionality and evaluated them based on three areas: conceptualization, research method, and interpretation/findings. Results: Our findings indicate that maternal health researchers have provided good descriptions of the interaction of systemic inequalities and have used analysis that allows for the examination of interlocking and mutually reinforcing social positions or systems. We find that improvement is needed in the areas of conceptualization, reflexivity, and understanding of power structure. Recommendations are provided in the form of a checklist to guide future research toward an impactful approach to addressing perinatal health disparities. Relevance: Our scoping review has implications for improving applied health research to address perinatal health disparities, mortality, and morbidity. Recommendations are given along with references to other tools, and a guidance checklist is provided to support scholars in creating an impactful approach to applying intersectionality in the goal of addressing maternal health disparities.

## 1. Introduction

The United States is the only developed country that has seen rapidly rising rates of maternal mortality and morbidity over the last 25 years [1]; despite having one of the most expensive healthcare systems in the world [2]. Severe maternal morbidity affects approximately 50,000 to 60,000 U.S. women each year throughout the entire perinatal period, and these numbers are increasing, with the most pronounced disparities being quantified by race, ethnic, socioeconomic, and insurance status [3,4]. Systemic inequality has contributed to these disparities. An increasing number of health researchers have begun to call for a more critical examination of structural issues embedded in social locations and healthcare systems that impact perinatal outcomes [5]. Intersectionality has become a popular research paradigm in population health research to explore structural inequality and bring about social change. However, there exists a lack of basic guidelines on how the intersectionality framework is applied in health sciences. There is an ongoing dialogue about how current research practices attend to the tenets of intersectionality to create real-world policy change and social impact [6]. Although there is no uniform approach to applying intersectionality, scholars have cautioned health researchers against common pitfalls, such as assuming “master” categories (e.g., gender, race, or class) to study [7,8]; summarizing; relying on an additive approaches [9,10]; linear analysis of the main effects based on separate and independent inequalities [11,12]; and lack of reflection on the role of the researchers and the impact of their identities/lived experiences on study design, implementation, and interpretation of research results [10,13,14]. The aim of our study is to conduct a scoping review of existing research to determine how past studies have applied the theory of intersectionality to explore maternal health disparities in physical health and mental health outcomes during the perinatal period (during pregnancy and up to one year of birth).

In addition, our work reviews existing studies and creates a checklist of requirements to help future researchers use intersectionality in maternal health research toward a more impactful approach. The findings provide insights on (1) how previous studies have used a critical approach to understand systemic inequality embedded in the realities of perinatal people, and (2) ways to improve the application of intersectionality toward the development of a checklist of guidelines for perinatal maternal health science. This insight further helps to produce important knowledge on structural inequalities and informs social change to address maternal health disparities in the perinatal period. In addition, our review strictly focuses on maternal physical and mental health outcomes during the perinatal period. We did this because we did not want to assume that the drivers of systemic inequity underlying perinatal health disparities are the same for infant health disparities. We also recognize that an intricate link exists between maternal and infant health disparities [15], especially in birth outcomes and this is a limitation in our approach to exclude all studies related to birth outcomes. Future studies should expand research inquiries on understanding both maternal and infant health disparities from a holistic perspective.

### 1.1. The Concept of Intersectionality

The initial expression of intersectionality started in the work of the Combahee River Collective (1977/1995), a group of Black feminists who described the simultaneous impact of both sexism and racism [16]. The concept of intersectionality already had deep roots in U.S. social and historical politics among the Black community when in 1989, Kimberlé Crenshaw, a scholar in the field of law, coined the term “intersectionality” [17]. Building upon critical race theory [18] and Black feminist thought [19], intersectionality provides a lens to understand lived experiences without reducing individuals to single characteristics. The approach assumes that social identities are not “inseparable and shaped by the interacting and mutually constituting social processes and structures that are influenced by both time and place” [6]. In other words, intersectionality accounts for how different systems of marginalization and privilege are based on one’s positioning to produce the unique experiences of the individual and their community.

While intersectionality was coined in the field of law, it has been widely applied to other fields. These include the fields of social science, humanities, business, and industrial organization. Within these areas, the intersectionality framework helps researchers to conceptualize and contextualize the impact of systemic inequity on different individuals and collective experiences. Intersectionality began to gain more traction in health science and population research over the past decade. Notably, it became a common research paradigm for furthering the understanding of the complexity of health inequities. This has helped health researchers to strive toward structural change to address disparities in the healthcare system [20].

Additionally, the intersectionality framework can be applied to many types of research approaches. For example, existing qualitative studies in health science have applied intersectionality to conceptualize and examine researchers’ and participants’ social positioning [10,21,22]. However, few research studies have focused on the physical health and mental health outcomes of perinatal people, with studies emerging only recently [23,24,25,26,27]. Notably, the work of Sen et al. [28] on gender inequality within a global economic and public health context covered the struggle of pregnant people in rural India from an intersectionality perspective. Our review did not find any studies that used mixed-method approach, which was a limitation of the existing literature. In summary, intersectionality is an important critical theory that holds the potential to bring about social change and address injustice through research. 

### 1.2. Current Study

The objective of our scoping review is to examine how existing research in maternal health has applied intersectionality in their study approach and conceptualization. We focus on how the application of intersectionality has been used to explore health disparities in maternal physical health and mental health outcomes during the perinatal period. The goal of our study is to learn about the strengths and limitations of existing works, and to provide recommendations regarding best practices, as well as a checklist for future research, to study maternal health disparities. In our study, we defined the intersectionality framework as a critical constructivist research paradigm with a focus on examining the interactions of multiple systems of oppression, power, and privilege that have shaped the lived experiences of people. To this end, the intersectionality framework also examines power positionalities of the healthcare system and all stakeholders from historical and cultural contexts in the field of perinatal health. To evaluate all studies included in our scoping review, we identified a list of research questions (Table 1) inspired by, and adapted from, colleagues [6,7]:

Based on our analysis and results, we considered additional questions to determine recommendations for future research. These questions included: what are the strengths and limitations of existing studies? How do we develop a checklist of guidelines for a research approach based on intersectionality to produce empirical evidence, balance research power among stakeholders, and translate learned knowledge into real-life impact rooted in social justice values?

## 2. Methods

The current scoping review followed the framework and method developed by Arksey and O’Malley [29], as well as recommendations from the work of Munn et al. [30]. Our steps included: (1) identifying the research question; (2) identifying relevant studies; (3) selecting studies; (4) charting the data; and (5) collating, summarizing, and reporting the results.

### 2.1. Identifying Studies

To identify relevant literature, we conducted a search across four search engines: Google Scholar, EBSCO, PsycINFO, and PubMed. Keywords used to identify studies included combinations of the following terms: “depress*” OR “*anxiety*” OR “*anxious*” OR “*mental*” OR “*psychological*” OR “*physical*” OR “*medical*” OR “*stress*” OR “*distress*” OR “*cardiovascular*” OR “*cardiomyopathy*” OR “*cardiac*” OR “*hypertension*” OR “*preeclampsia*” OR “*diabetes*” OR “*obesity*” OR “*infection*” OR “*hemorrhage*” OR “*eclampsia*” *OR* “*abruptio placentae*” OR “*placenta previa*” OR “*asthma*” OR “*respiratory*” OR “*mortality*” OR “*morbidity*” OR “*comorbid*” AND “*maternal*” OR “*perinatal*” OR “*pregnancy*” OR “*partum” OR “*antenatal*” OR “*postnatal*” OR “*prenatal*” AND “*intersectionality*” OR “*positionality*” OR “*positioning*” OR “*reflexivity*”. We used intersectionality and positionality as synonyms in our search strategy to achieve a broader search. Duplicates, dissertations, theses, and/or studies of insufficient relevance were excluded. We included studies with original research that were published in English, and which strictly examined perinatal health outcomes through an intersectional perspective.

### 2.2. Literature Selection

**Figure 1:**
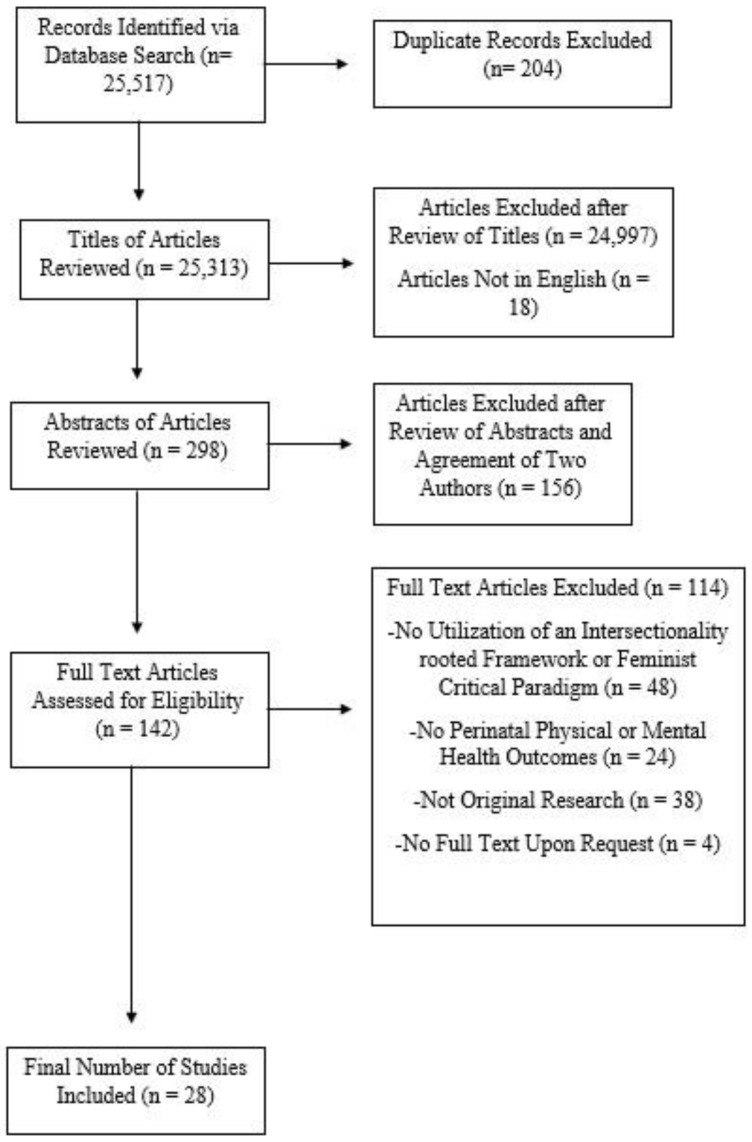
Flowchart of the search and criteria for inclusion in our scoping review.

Initially, our search produced 25,517 records. We excluded 204 duplicate articles, and then further excluded articles if their titles did not allude to a focus on perinatal maternal health outcomes. After excluding these titles, 298 pieces of literature remained. Both authors read the abstracts of all remaining articles to determine eligibility. Studies of birth outcomes that focused on preterm birth and low-birth weight were then excluded, as these outcomes overlapped with infant health outcomes. Eligibility criteria for study inclusion included: (1) use of an intersectionality framework in the research approach/design, (2) inclusion of at least one maternal physical or mental health outcome during the perinatal period (from pregnancy to up to one year of birth), (3) written in English, and (4) inclusion of original research and not a review or opinion. After determining eligibility, a total of 32 research papers remained. Certain articles were not directly accessible because they were locked behind a paywall or did not have the full-text version. For articles that were not directly accessible, we attempted to access them through an interlibrary loan or by contacting the authors directly. After discarding inaccessible articles, 28 eligible, original articles remained. Our final sample included 28 published original research articles with a total sample of 9,856,042 participants (Figure 1).

### 2.3. Charting and Summarizing Data

We first created a standardized worksheet to evaluate the 28 published research articles and to summarize the data. This worksheet included the findings of each article and the research questions used to evaluate each study individually. We reviewed, analyzed, and summarized the results. To provide a cohesive analysis of the studies, we utilized a standardized form to evaluate the studies based on each research question. There was a column for each research question, and any details about each study that directly answered the research question were extracted and charted under each column by both authors. We determined that if a research study included at least one detail or one step that directly answered our research question, it was considered “satisfied” for our evaluation. Each author evaluated each study independently and we met together seven times to discuss differences and to reach a consensus. Differences in our evaluation were discussed and each reviewer spent additional time revisiting each study. Consensus was reached based on an iterative process of reviewing and evaluating until all differences were resolved. We summarize our results in two tables: Table 2 displays the characteristics of each study, which Table 3 shows a summary of the findings based on our research questions. A checklist is presented in Table 4 to guide researchers on how to apply intersectionality in future studies.

## 3. Results

We included 28 published articles conducted on perinatal health outcomes. The majority of the studies (*n* = 16) were conducted in the United States and 12 studies were conducted in other locations (Israel, India, Australia, Romania, North America, Vietnam, Tunisia, Ireland, South Africa, Europe, and England). Out of these 28 original research articles, 11 used quantitative methods and 17 used qualitative methods. For this review, we were unable to find any mixed-method studies. For additional study characteristics, please refer to Table 2. In the following sections, we present our results which are based on the three main areas outlined in Table 1: conceptualization, research method, and interpretation/findings. We also display the quantitative and qualitative studies separately for a clear demonstration of the results. 

### 3.1. Use of Intersectionality in Study Conceptualization

We examined studies for whether they discussed the effects of systems of inequality (i.e., racism, sexism, colonialism, etc.) on perinatal health outcomes. This was done in addition to examining the interactions of demographic and social identities among the studied population (Table 1, research question 1). We also assessed whether studies framed their research topic within the historical and current cultural, societal, and/or situational context (Table 1, research question 2). For example, a study on rural Aboriginal communities included the historical, social, and economic contexts of a rural population situated within the broader context of global economics and neocolonialism [20]. Another example study on gendered racism and maternal mortality focused on how the historical and current context of medical practices and reproductive rights within a state framed the experiences of Black, pregnant women [26]. By considering the history of reproductive rights in the US at the state level, Patterson and colleagues [26] accounted for the socio-historical context and structural power relations that shaped perinatal health disparities. 

Overall, all 28 studies discussed the effects of multiple systems of oppression and structural inequality on the health outcomes of the perinatal population (Table 1, research question 1). Five of the quantitative studies used social and demographic variables as proxies to study the interaction of multiple systems of inequality on perinatal health outcomes. One example is Albright et al. [23], which used demographic variables such as age, race/ethnicity, annual income, state of residence, and veteran status as proxies by comparing them against mental distress, current cigarette use, and alcohol consumption of participants. Few studies (*n* = 5) included structural and contextual variables, such as poverty levels, crime rates, or state policies on reproductive care, to examine macro and meso-level social factors that impacted intersectional positions. For example, Seng et al. [37] included indicators that reflected both structural inequality factors, such as low education levels and poverty, and contextual factors, such as crime rate and trauma exposure, to operationalize meso- or interpersonal-level factors. Nine studies also included self-reported scales of perceived discrimination, such as gendered racial discrimination or everyday discrimination experiences, to account for the lived exposures of systemic inequities and social processes. For example, Clarke et al. [31] used subscales to measure stressors such as gender role strain, racial stereotyping, and racism/sexism in the workplace. Our findings also showed the most common methods in assessing intersectionality effects were regression with interaction terms, models using stratification, and categorized intersectional positions. These findings aligned with three systemic reviews to show that this was also a pattern across health science and public health research [13,60,64]. 

On the other hand, all identified qualitative studies included open-ended questions to examine the interaction of systems of marginalization on the lived experiences of participants. In summary, across both quantitative and qualitative studies, a total of 25 studies that framed their research topics within the current cultural, societal, and/or situational context. Only 18 of these 25 studies described the historical context that framed their research population (fulfilled both research questions 1 and 2 in Table 1). In summary, 18 papers (approximately 64%) fulfilled both research questions (Table 1, questions 1 & 2) in this section (see Table 3 for full analysis).

**Quantitative Research.** Among the quantitative research studies, 5 of the 13 studies fulfilled all research questions (Table 1, questions 1 & 2). The remaining 8 studies had a tendency to examine the interactions of systems of inequalities but did not frame the research question within the current cultural, societal, and/or situational context (see Table 3). One excellent example of a study that fulfilled both questions was by Rosenthal and Lobel [27]. This study measured the lived experiences of gendered racism using a scale rather than relying on interaction analysis of demographic variables to account for lived exposure of social and structural inequities. Furthermore, they analyzed historically rooted stereotypes that influence the experiences of oppression by perinatal Black and Latina women. For example, they discussed stereotypes related to sexuality and motherhood such as the “welfare queen” and “sexual siren” [27]. Rosenthal and Lobel [27] also extended their discussion to describe the history of medical experimentation on the studied population within the United States. In particular, they looked at the impact of forced sterilization of Black and Latina women. They were able to provide both a historical and current background to contextualize the health disparities experienced by perinatal Black and Latina women. Another example of a quantitative study that fulfilled both questions was the work of Patterson et al. [26]. This study framed their work within a historical context by taking into account the political environment in terms of reproductive justice within different states of the United States. They discussed and examined the supportability on reproductive rights within each state and the impact of state policies on maternal health outcomes.

**Qualitative Research**. There were a total of 17 qualitative studies. All of them met the criteria for fulfilling research question 1 from Table 1. Thirteen of them fulfilled question 2 by framing their research topics within the current and historical context (see Table 3 for full analysis). A good example was a study by Mehra et al. [48], where they discussed gendered racism within the economic, reproductive rights, and social justice contexts. They described historical and current stereotypes that stigmatized Black motherhood, such as the sexist, racist presentation of the “welfare mother” and the sexually aggressive “Jezebel” [48]. This study was able to explore both the interactions of social positioning and systemic forces within the social and historical contexts of the lived experiences of Black pregnant women.

### 3.2. Use of Intersectionality in the Research Method

For research methods, we evaluated the studies based on whether the authors mentioned power differences or dynamics that contextualized perinatal health disparities and the role of researchers within the socio-historical hierarchies (Table 1, research question 3). We also looked for studies that actively addressed the power differential, such as engaging key stakeholders in their research design and implementation to address the power differences and to include the voices of stakeholders when making important decisions (Table 1, research question 4). Along this line, we assessed whether researchers reflected on their identities, their roles in the research hierarchy, and how these factors impacted their decisions in designing, implementing, and analyzing the study. Lastly, we evaluated studies based on whether they used a multidimensional analysis to study intersectionality (Table 1, research question 5). Using a method that allows for multidimensionality is important when working with an intersectionality framework because of the impacts of the interlocking social positions of power. Therefore, it is impossible to pinpoint which social identities or processes are the most salient at any given time.

Our findings showed that the majority of studies (*n* = 19) did not discuss the social power relations that contextualize health disparities and their roles within the research hierarchy (Table 3). Seven out of the 28 studies engaged with stakeholder groups to allow for power sharing in determining their research approach and implementation. Furthermore, another 7 of 28 studies accounted for the impact of researcher positionality and/or lived experience on data collection and analysis. Additionally, almost all of the studies (*n* = 23) used data collection and analysis to allow a multidimensional understanding of intersectionality rather than relying on an additive approach. However, given that we only evaluated studies based on reflexivity and consideration of addressing power dynamics, only 4of the 28 studies (13%) in our scoping review met the criteria for satisfaction in this section (Table 3).

**Quantitative Research**. Within the quantitative studies, none met all of the criteria to satisfy our research questions in this section (Table 1, questions 3, 4 & 5). Most studies (*n =* 10) did not discuss the context of social and structural power or acknowledged the power differential in their research approaches. Further, most studies did not provide a positionality or reflexive description for their chosen methods and resulting interpretation. One quantitative study did include a stakeholder group to account for power imbalances during research design and implementation. For example, Vedam et al. [38] incorporated a stakeholder group of community agency leaders, clinicians, and researchers to develop research questions and adapt survey instruments for their study. This consultation allowed the researchers to work with stakeholders to develop survey items that best captured their unique perspectives and lived experiences. While Vedam et al. [38] did not discuss their reflexive process, the inclusion of a stakeholder group reflected their attention to issues of power within their study.

Furthermore, based on the recent metaanalyses of colleagues [13,60,64], quantitative methods are still adapting and developing to accommodate the core tenets of intersectionality. Our finding aligned with those of Bauer and colleagues [13] and Guan and colleagues [60] which showed that the majority of studies in our review used regression models with interaction terms to identify unique impacts of interlocking social positions and power. Although most statistical methods have the potential to explore intersectional effects, scholars suggest future researchers to provide a rationale for why they select a specific method or approach, to clarify underlying assumptions, biases and limitations of their methods, and explain how their chosen approach can be interpreted in the context of social and structural power [13,60]. We highlight here the most common methods to study intersectionality [13,60,64] in health science research: (1) regression with interaction terms (e.g., linear and other models with identity factors, multiplicative and other models with logit or log links, ANOVA-based methods, chi-square, *t*-tests), (2) additive and multiplicative scaling, (3) cross-classified variables, and (4) stratification. In addition, leading intersectionality scholars have asserted that the assessment of additive scale interaction is the most relevant for health-related research because it is a representation of intersectional multiplicativity [8,9]. Additive scale interaction is also noted to be helpful and more informative for clinical decision making and public health interventions [60,65].

**Qualitative Research.** Six of the 17 qualitative studies discussed the context of social and structural power as well as utilized a stakeholder group to account for power differences and to reflect upon the impact of the lived experiences of the researcher on the process of data collection and analysis (Table 3). Additionally, 13 studies incorporated a method to study identities at the multidimensional level (Table 1, question 5). One example was a study by Taylor et al. [52], which engaged a group of community members with lived experiences in data analysis and interpretation of results. This allowed for the voices of both researchers and stakeholders to be heard, and more importantly, be included in the data analysis and interpretation stages. This study also provided critical reflections on the experiences of the first author working in perinatal mental health and the experiences of the last author regarding the utilization of perinatal mental health. Lastly, Taylor et al. [52] framed their interview questions without prioritizing specific identities/experiences so that participants could describe their lived journey based on what they thought was important. For example, the research team asked questions such as: “Can you start by telling me a bit about your pregnancy?” and “How were things after birth?” [52]. The questions were framed so that the participants could reflect and describe social identities or processes that felt most salient to them. 

### 3.3. Use of Intersectionality in the Interpretation and Findings of the Study

We determined whether the studies provided the context of structural barriers or inequity on the physical and mental health outcomes of perinatal women (Table 1, research question 6). Similar to the research method, we also assessed whether the researchers reflected on how their lived experiences and identities influenced the interpretation of the results (Table 1, research question 7). Our results showed that 25 of the studies mentioned the impact of systemic inequity on perinatal health outcomes. In contrast, only 6 out of 28 studies provided lived experience reflections from the researcher and how they potentially impacted the interpretation of the results. Only 5 studies satisfied both evaluation criteria in this section (Table 3).

**Quantitative Research.** None of the quantitative studies fulfilled both questions for this section (Table 1, questions 6 & 7). Despite this, all of the studies discussed the impact of systemic inequity on the health outcomes of the perinatal population. One study described forms of discrimination at the individual level, such as verbal abuse during provider interactions, disregard for autonomy, and rights to medical information [38]. Others described oppression at the macro level, including the impact of unequally distributed wealth, lack of social support, barriers to health care access, and barriers to upward mobility [36,66].

**Qualitative Research**. Only five of the qualitative studies fulfilled both research questions in this section (Table 1, questions 6 & 7). For example, Altman et al. [24] discussed how structural factors, such as historically racist and patriarchal systems withing the United States impacted patient–provider communication and healthcare delivery. Additionally, they provided a statement on how the lived experiences of the authors influenced the data interpretation. The researchers reflected upon how their racial identities and positions as healthcare providers shaped their interpretation of their findings.

## 4. Discussion

Maternal mortality and morbidity are currently critical issues in the United States, with alarming disparities among perinatal minoritized populations, who face multiple systems of oppression within the existing social structure of power. Intersectionality has the potential to move health science toward reducing maternal mortality and morbidity, addressing disparities, and transforming social hierarchies responsible for inequality [20,60]. As more maternal health researchers continue to develop methods and apply intersectionality to address perinatal inequities, our findings shed light on areas of strength and places for improvement so that future research can apply intersectionality in more impactful ways and strive toward improving perinatal health outcomes. Importantly, our review develops a checklist of considerations (see Table 4) to improve research approaches toward fostering systemic change and challenging current social and structural power that contribute to health disparities.

### 4.1. Conceptualization and Research Goals

Most of the studies reviewed here described current contexts that shape systemic disparities in both their conceptualization and interpretation sections. However, few of the studies discuss the socio-historical hierarchies that are responsible for power differential and current disparities and injustices. Similar to the explanations provided by Hankivsky [6] and Hancock [8], our findings demonstrate the common issue of inadequate conceptualization in research that uses intersectionality. This is especially prevalent when the studied categories are rooted in social processes and construction within existing power relations. To address this limitation, we urge researcher to follow the suggestions of intersectionality colleagues [6,13,60] to: (1) justify the use of intersectionality in an analytical approach, (2) explain how intersectional effects are examined through the chosen method, (3) draw connections between biological, historical, and social processes to help advance understandings of specific categories and illuminate why social context and power relations are so important for the construction of gendered health outcomes. An excellent example of the construction of gendered health outcomes that would be useful to reference is that from MacDonald et al. [45], which considered how pregnancy and the desire to be pregnant are seen as inherently feminine in Western culture, which could impact the experience of transmasculine individuals who desired to be pregnant.

Furthermore, we recommend researchers review our checklist along with other guidelines of conceptualization from leading intersectionality scholars [6,7,11,54,55]. For example, McCall [54] describes the differences between intracategorical approaches (i.e., focus on complexity of experience within a particular social position or intersection), intercategorical approaches (i.e, focus on heterogeneity across a range of intersections), and anticategorical approaches (i.e. the critique of rigid social categorization itself). Bauer et al. [13] concluded that most research works were intercategorical, which focused on describing inequalities across intersections. There needs to be more expansion in terms of other types of approaches because repeatedly documenting inequalities, even in finer intersectional detail, can serve to reinforce ideas of inherent differences between groups rather than to point towards actionable solutions [9]. Intersectionality is developed and rooted in Black feminist activism, so its main principle ties to the goal of advancing social justice [60,62]. Researchers should not disconnect, dilute, or depoliticize its original function in social change. Researchers should begin to incorporate social justice values and agendas into their conceptualization and research design [62]. The goal of intersectionality research should work toward identifying clear and implementable solutions to advance health equity and social justice [13,60].

### 4.2. Review of Research Methods

Our analysis shows that all included research studies recognized the impact of the interaction of systemic forces and identities on perinatal physical health and mental health outcomes. Almost all of the studies also used data analysis approaches to account for the interaction of multiple social systems and identities. However, the majority of studies focused on social identity factors rather than structural factors embedded in existing social processes (i.e., racism, sexism, or ableism). These findings are consistent with several recent systemic reviews on quantitative studies [13,60,64], which discuss how research approaches often do not contextualize their results within broader systems of power and oppression. On the other hand, qualitative studies have done a better job of acknowledging power structure and considering power differential in the research process. In summary, selection of research method highlights the researchers’ assumptions and biases. This process needs to continue to be developed because intersectionality does not originate as an empirically testable framework. Scholars routinely discuss the absence of guidelines and challenges regarding the appropriateness of different methods to capture the complexity of the tenets of intersectionality, especially when researchers overlook factors related to social power [13,57,60].

Many scholars across disciplines caution about the methodological issues of misrepresentation and inaccurate interpretation of demographic intersectional effects, especially in quantitative studies [13,64,67]. In a recent systemic review, Bauer and colleagues [13] critiqued common quantitative approaches, which included regression models with interaction terms between two or more social positions. These interaction terms often did not clearly distinguish between regression analyses of intersectional inequalities versus causal effects. Neither did they provide the rationale behind choice of multivariable analyses. We agree with colleagues [13,60], and recommend researchers to do more reflexive reflection about their chosen method including describing their rationale, limitations, or biases/assumptions inherent in their research, as well as how intersectionality framework is applied within their approaches. The process of making these explicit may also drive a deeper engagement with ideas in foundational and methods literature and require researchers to question the power structure embedded in the socio-historical construction of their categories as well as the limits of categorization [13]. We recommend researchers also review guidelines for quantitative research methods through the work of Guan et al. [60], Phillips et al. [64], Bauer et al. [13], and qualitative methods through the work of Bowleg [10,57], and Choo and Ferree [12].

### 4.3. Accounting for Social and Structural Power Relations toward a Stakeholder-Engaged Approach

Our results demonstrate that few studies account for broader social power relations within existing hierarchies. Notably, few researchers described their reflexive process on how their lived experiences and identities impacted their selections of research design, implementation, analysis, and interpretation. This finding is not new and is a part of the ongoing discussion about challenges in conducting intersectionality research and the importance of exploring epistemological assumptions made by the researcher when forming the methodology [8,10,14,68]. Leading intersectionality scholars [6,13,14,55,59,60] have urged researchers to reflect on their decisions during the research process and on power relations that are responsible for health disparities so that researchers are not replicating the patterns of exploitation and marginalization of studied populations. Previous tainted medical projects, such as the Tuskegee syphilis experiment in the Black community, the 1956 birth control trial among poor uneducated Puerto Rican women, or the forced sterilization of Black and Latina women, for the basis for mistrust and power differences between the studied population and health scientists. We take the stand that health science research should begin to address the power dynamics and be accountable in producing knowledge to make impact and challenge existing social and structural power relations, which contribute to current health disparities and injustices. 

As a part of recognizing the power dynamics and striving toward larger social and political change, researchers need to pay attention to the power and the assumptions that they hold with regard to the research process. To this end, community-engaged, patient-engaged, or stakeholder-engaged research has been documented to be valued approaches due to their effectiveness in reducing health inequities, creating impact, and challenging the existing power hierarchy [69,70]. Findings from 126 studies funded through the Patient-Centered Outcomes Research Institute (PCORI) showed that contributions from stakeholders could be incorporated in all phases of the research process. This includes conceptualization, design, intervention, recruitment/retention, data collection/analysis, and dissemination [69]. Forsythe and colleagues [69] summarized the engagement effects into four themes: (1) acceptability (research agenda, goals and interventions were well received and aligned with stakeholder values and needs), (2) feasibility (roadblocks to effective implementation could be eliminated), (3) rigor (research choices that minimize bias and enhance data quality), and (4) relevance (research that creates impact for the community, patients, and providers). One study example was Taylor et al. [52], which used a lived experience group to create and revise research questions. They also collaborated with this group to draw out themes during data analysis to ensure that the experiences of women with perinatal depression were accurately reflected and interpreted by the stakeholders’ lived experiences. By doing this, their team began to address the power imbalance between researcher and participant. This allowed the study to produce results that were relevant to the population of interest as well as making real-world impact in the community as a part of the data dissemination process.

### 4.4. Checklist to Apply Intersectionality Framework

Table 4 presents our checklist, which builds upon previous research recommendations and methods that focus on person-centered and system-centered approaches as well as community-engaged partnerships through trusted relationships, power sharing, and transparency. When possible, we recommend researchers attempt to incorporate all steps of our checklist and consider all guiding questions in Table 3. We recognize that researchers do encounter limitations in their research approach based on their research questions and the types of data available. These limitations can be exacerbated by limited opportunities to engage stakeholders for secondary analysis. We, however, support the recommendation that intersectionality scholars need to ensure future researchers should be explicit regarding the explanation of aims, hypotheses, limitations, and application of intersectionality within their research approaches. These criteria recommend future researchers discuss the cultural, societal, and/or situational contexts of the intersectional positions that they study. The researchers are encouraged to describe the systems of oppression and power that shape/contextualize health outcomes, and justify for the selection of analytical method to capture intersectional effects. We recommend researchers to engage stakeholders whenever the design allows even at the last stage of the dissemination process. Lastly, although we do not think all research designs require reflexivity, we urge researchers to continuously reflect on where they are located in the hierarchy so that they do not perpetuate more experiences of marginalization in the research process [59]. By taking these steps, researchers will already begin to make an impact on addressing systemic health inequalities and injustices, especially when working with minority communities. Moreover, the intersectionality framework is rooted in social justice and activism. Hence, future researcher should be intentional in its research goal toward identifying clear and implementable solutions which can be used to advance health equity and challenge existing social hierarchies [60,62].

To the best of our knowledge, our work is the first scoping review in the field of perinatal health and mental health which looks at the application of the intersectionality framework. Our review provides a comprehensive understanding of how existing research has incorporated intersectionality in conceptualization and research design in addressing perinatal mortality, morbidity, and disparity. We recognize that a limitation of our study is that we only used published studies written in English. All of the included studies had perinatal women’s physical and mental health outcomes while excluding preterm birth and low-birth weight studies. This represents a limitation in our inclusion criteria as we recognized that birth outcomes were critical to both maternal and infant health disparities. Furthermore, we could not conclude that the results in our review would apply to the fields of infant and child health. We also included studies that only incorporated intersectionality in their conceptualization and research design, so we could not comment on the rest of the research literature on perinatal health outcomes that did not use the intersectionality framework. Additionally, four studies were excluded after all efforts had been made to gain access to them. Our sample also does not include any mixed-method studies, which demonstrates that this approach is lacking. Future research needs to explore these mixed methods more, as mixed methods have the potential to capture more tenets of intersectionality [6]. 

## 5. Conclusions

To address rising health disparities, mortality, and morbidity among perinatal people in the United States, we urge researchers to extend their approaches by incorporating intersectionality and following our checklist to create more impactful knowledge that brings about more meaningful social change. It is our goal that our checklist helps researchers by giving them tangible steps in methodology, and it is a place where resources are consolidated so that researchers can continue to expand their methods. Our overarching goal is to further the production of important knowledge on structural inequalities, which can then inform policy change and create a more meaningful impact in addressing health disparities in the perinatal population. 

## Figures and Tables

**Table 1:** Research Questions.

**Conceptualization**
1. Did the study examine the interaction of multiple systems of oppression and privilege on the physical and mental health outcomes of perinatal women?
2. Was the research topic framed within the historical and current cultural, societal, and/or situational context? (Adapted from [20])
**Research Method**
3. Did the researchers account for power differences in socio-historical hierarchies? Did they attend to the power differential by actively involving key stakeholders in the process of research design and implementation?
4. Did the researchers reflect on how their lived experiences and identities impacted the process of data collection and analysis? Were they being reflexive?
5. Did the study use a method that allowed for a multidimensional or multilevel examination of intersectionality without relying on an additive and linear analysis?
**Interpretation/Findings**
6. Did the researchers discuss the impact of systemic inequality on the health and mental health outcomes of perinatal women in their findings?
7. Did the researchers reflect on how their lived experiences and identities impacted their interpretation of the results?

**Table 2:** Study Characteristics.

Author(s), Year	Country	Population Characteristics			Races/Ethnicities Included	N	Aim/Objective of Study	Outcomes
		Age	Pregnancy Status	Other Relevant Characteristics				
**Quantitative Studies**								
Albright et al. [23]	USA	>18 yrs	Currently pregnant	Veterans and non-veterans	White, racial/ethnic minority	6101	To determine if pregnant veterans of an ethnic/racial minority have a higher likelihood of engaging in binge drinking	The prevalence of binge drinking was highest for racial/ethnic minority veterans (17.42%) when compared with White veterans (5.34%), non-veteran racial/ethnic minorities (4.05%), and non-veteran Whites (3%).
Clarke et al. [31]	USA	18–40 yrs	8–14 wks gestation	Only included singleton pregnancies	African American	485	To assess if gendered racial stress adds a dimension to prenatal stress that is independent from perceived stress and stressful life events.	Contextual gendered racialized stress is a distinct dimension of psychosocial stress for pregnant Black women and is associated with increased depressive symptoms.
Daoud et al. [32]	Israel	16–48 yrs	6 wks–6 mths postpartum	Immigrants and non-immigrants	Palestinian-Arab or Arab Jewish	1128	To determine if there is an association between experiences of discrimination and postpartum depression	Multiple forms of discrimination and ethnic discrimination had a strong association with postpartum depression for nonimmigrant Jewish mothers and Palestinian-Arab mothers, but not for immigrant Jewish mothers.
Hailu et al. [33]	USA		≥20 wks gestation	From California	White, Black, Hispanic, Asian/Pacific Islander, Native/Mixed	9,806,406	To examine the risk of severe maternal morbidity through an intersectional lens.	Severe maternal morbidity increased with age across the categories of education and neighborhood deprivation. Specifically, this association was the most significant among Black women.
Hartnett et al. [34]	USA	15–44 yrs	Pregnant in the last 5 years	Included four sexual orientation categories	White, Black, Hispanic/Latina	15,163	To examine how smoking habits during pregnancy are influenced by race/ethnicity and sexual orientation	Sexual minority status is association with a higher likelihood of smoking during pregnancy. Black and Latina women had lower odds of smoking than White women when control variables were included.
Misra et al. [35]	USA	12–43 yrs	22–28 wks gestation or postpartum while in hospital	Lived in Baltimore City, Maryland	Black	832	To determine if the social/psychosocial factors that impact Black women influence their risk of preterm birth	Lifetime experiences of racism had no overall effect on preterm birth, but women with higher stress scores had a stronger adverse reaction to experiences of racism.
Patterson et al. [26]	USA	15–50 yrs	Past pregnancy related to mother’s death	From all 50 states	Black, White, racial/ethnic minority	6900	To determine if women of different races/ethnicities experience differential weathering and risk of maternal mortality due to state supportability of reproductive rights	Black women’s maternal mortality rates were typically double those of White women, even when controlling for age and state supportability.
Rosenthal & Lobel [27]	USA	≥18 yrs	Currently pregnant and/or had at least one child	From New York City or Long Island	Black, White, racial/ethnic minority, Latina	343	To determine if the experience of stereotyped, gendered racism affects the healthcare outcomes of pregnant women of a racial/ethnic minority	Black and Latina women reported a greater frequency of stereotype-related gendered racism and greater birth control related mistrust than White women.
Sen & Iyer [36]	India	Unspeci-fied	Currently pregnant and postpartum women, and nonbirthing men/women	Used caste, income, and gender as main identifiers	Racial/ethnic minority, mixed race	15,358	To determine if socioeconomic ordering impacts how different groups secure healthcare treatment	For non-poor households, women who did not earn an income had a better chance of continued treatment. However, in terms of spending, non-poor women spent about the same as poor men.
Seng et al. [37]	USA	≥18 yrs	>23 wks gestation	All participants were expecting their first child.	White, Black, Asian/Pacific Islander, Native American/Alaskan Native, Hispanic, Middle Eastern	647	To model an intersectionality framework in an interpersonal, structural, and contextual way when researching marginalized identities.	At the structural level, Black women were more disadvantaged than White or Asian women. At the contextual level, Black women were more likely to live in zip codes with a higher crime rate and had the greatest number of trauma exposure.
Vedam et al. [38]	USA	25–35 yrs	Women who experienced at least one pregnancy from 2015–2016 (includes currently pregnant)	Majority of participants used a midwife for prenatal care	Black, White, racial/ethnic minority, Asian/Pacific Islander, Hispanic, Indigenous	2138	To determine if inequity and mistreatment are more prevalent in the healthcare experiences of pregnant women of a racial/ethnic minority	17.3% of participants experienced at least one form of mistreatment. Likelihood of being mistreated was lower for those who had a vaginal or community birth, utilized a midwife, were White, were multiparous, and were older than 30 years old.
**Qualitative Studies**								
Altman et al. [24]	USA	≥18 yrs	6 wks–1 yr postpartum	Must self-identify as a person of color	Black, Hispanic, Indigenous, Latina	22	To determine the perception and understanding of interactions with providers within the context of maternal care	An established patient–provider relationship and levels of privilege or marginalization impacted how providers chose to share information.
Amroussia et al. [39]	Tunisia	19–43 yrs	Had given birth at some point	All participants were single mothers	Tunisian and Algerian	11	To examine single mothers’ self perceptions and experience receiving healthcare during childbirth.	Participants reported feeling shame and regret for being a single mother as well as experiencing abuse and disrespect when receiving healthcare.
Andalibi et al. [40]	USA	>18 yrs	Experienced pregnancy loss in the last 2 years	Had to identify as LGBTQ+	White, Black, Latinx, Multiple Races, Human	17	To explore the benefits and challenges of utilizing LGBTQ specific and nonspecific online pregnancy loss spaces.	A shared sense of identity and experience was associated with a supportive community online, but larger forums or groups still lacked representation and/or visibility of experiences and identities.
Chadwick [41]	South Africa	18–42 yrs	Had given birth at some point	Specifically looked at low income women	Black	35	To explore obstetric violence and how it impacts women’s agency during birth.	Obstetric violence functioned as a form of discipline that shaped the actions of women during labor.
Daoud et al. [42]	Israel	24–41 yrs	1 yr postpartum	Sample included hospital directors, midwives, and birthing women	Palestinian-Arab or Jewish	76	To examine what mechanisms drive the racial maternal separation of birthing mothers	Although many hospital directors disagreed with the practice of racial maternal separation, there were not enough policies in place to prohibit it.
Dove-Medows et al. [25]	USA	18–36 yrs	8–29 wks gestation and had a singleton pregnancy	Took a convenience sample of the first 18 cases of a larger study	Black	18	To explore the experience of racism that pregnant Black women face and how they manage it	Experiences of racial discrimination happened in different contexts, and in order to manage racism, participants often used a shielding technique.
Huschke [43]	Ireland	25–47 yrs	Pregnant or gave birth within the last 12 months	Participants were from the Midwest region of Ireland	Irish or Non-Irish	23	To explore women’s experiences of mental health while pregnant, during birth, and postpartum.	Women were rarely given different courses of action during their healthcare experiences or were told to do nothing.
LeMasters et al. [44]	Romania	24–39 yrs	Had given birth at some point	Sample also included healthcare providers	Roma, Romanian, Hungarian	61	To explore the experience of pregnancy for rural Romanian women and their interactions with the healthcare system	Transportation and cost to healthcare facilities was a barrier for most pregnant Romanian women who lived in isolated communities.
MacDonald et al. [45]	North America, Australia, Europe	Unspecified	Carried to term, currently pregnant, or had a miscarriage	All participants are transmasculine individuals	White, Black, Non-Hispanic	22	To explore the pregnancy and birthing experiences of transmasculine individuals.	During interviews, participants mentioned how their pregnancy related to the gender binary.
Mantovani & Thomas [46]	England	16–19 yrs	Were mothers or currently pregnant	Focused exclusively on teens looked after by the state	Black	15	To explore the experiences of pregnant Black teens who are looked after by the state	Participants felt as though they had been stigmatized by their providers when their pregnancies were discovered.
McLemore et al. [47]	USA	≥18 yrs	Currently pregnant or gave birth	Had medical/social risk factors for preterm birth	Black, Hispanic, mixed race	54	To explore maternal healthcare experiences and determine what contributes to the stress of pregnant people	Patients described their perinatal healthcare experiences as disrespectful and stressful due to insufficient social support and a lack of informational knowledge.
Mehra et al. [48]	USA	21–45 yrs	Currently pregnant	Lived in New Haven, Connecticut	Black	24	To explore the experiences of gendered racism during pregnancy	Racialized pregnancy stigma was experienced in the form of stereotypes that contributed to the devaluation of Black pregnancy and motherhood. These assumptions were encountered in multiple contexts regardless of socioeconomic or marital status.
Nguyen et al. [49]	Vietnam	25–45 yrs	Given birth in the last 3 yrs	Participants had physical disabilities that effected mobility or functioning of the hands and/or arms	Vietnamese	29	To explore how women with physical disabilities experience pregnancy.	Many participants felt happy or excited when they discovered they were pregnant. They also experienced some ambivalence and mixed range of emotions including anxiety, fear, self-doubt, and uncertainty; specifically when wondering how their disability would impact their pregnancy.
Staneva et al. [50]	Australia	22–46 yrs	Confirmed pregnancy that progressed to the second trimester	Participants were excluded if they experienced severe suicidal ideation	White Australian, White New Zealander, White North American, South East Asian	18	To explore how antenatal women interpret and view their experience of psychological distress.	Women who experienced symptoms of depression or anxiety during the antenatal period struggled to fit their narrative within that of the concept of the “good mother”.
Stevens et al. [51]	USA	19–43 yrs	Within the perinatal period	All participants were referred for mental health treatment at an outpatient psychotherapy unit	Black, Hispanic, White	67	To examine the effectiveness of coordinated perinatal mental health care.	Participants had high motivation for treatment. African American women were the most engaged in treatment, as they had the lowest rate of early treatment termination and highest average number of attended sessions.
Taylor et al. [52]	England	≥16 yrs	6–9 mths postpartum	Diagnosed with perinatal depression	Black, White, racial/ethnic minority, Palestinian-Arab or Arab, Mixed Race	14	To determine if the intersection of certain identities influences the prevalence of isolation and marginalization when regarding perinatal depression	Feelings of depression were often connected to a feeling of dislocation of identity and feelings of being unsupportive. Fear of being judged as an inadequate mother prevented participants from reaching out.
West & Bartkowski [53]	USA	18–40 yrs	Must have given birth within the last 5 years	Women who gave birth in- and outside of a hospital setting	Black	35	To explore the differing experiences of Black women birthing inside a hospital setting and outside a hospital setting	Power asymmetry in patient–provider relationships exists both within and outside the hospital setting, but out-of-hospital births have more covert power dynamics.

**Table 3:** Evaluation of Study Using Intersectionality.

	Research Question 1: Examined the Interaction of Systems of Inequalities in Perinatal Outcomes?	Research Question 2: Framed within the Current Cultural, Societal, and/or Situational Context?	Research Question 3: Accounted for Power Differences and Actively Involved Key Stakeholders?	Research Question 4: Were Researchers Being Reflexive in Data Collection and Analysis?	Research Question 5: Used a Method to Allow for Multi-Dimensional or Multilevel Examination?	Research Question 6: Discussed the Impact of Systemic Inequality in the Findings?	Research Question 7: Were Researchers Being Reflexive in Interpretation of the Result?
**Quantitative Studies**							
Albright et al. [23]	YES	YES, but only current context	NO	NO	YES	YES	NO
Clarke et al. [31]	YES	YES, but only current context	NO	NO	YES	YES	NO
Daoud et al. [32]	YES	YES	NO	NO	YES	YES	NO
Hailu et al. [33]	YES	YES, but only current context	NO	NO	YES	YES	NO
Hartnett et al. [34]	YES	YES	NO	NO	YES	NO	NO
Misra et al. [35]	YES	NO	NO	NO	YES	YES	NO
Patterson et al. [26]	YES	YES	NO	NO	YES	YES	NO
Rosenthal & Lobel [27]	YES	YES	NO	NO	YES	YES	NO
Sen & Iyer [36]	YES	YES	NO	NO	YES	YES	NO
Seng et al. [37]	YES	YES, but only current context	NO	NO	YES	YES	NO
Vedam et al. [38]	YES	YES, but only current context	YES	NO	YES	YES	NO
**Qualitative Studies**							
Altman et al. [24]	YES	YES	YES	YES	YES	YES	YES
Amroussia et al. [39]	YES	YES	NO, but acknowledges power differentials	NO	YES	NO	YES
Andalibi et al. [40]	YES	YES	NO, but acknowledges power differentials	YES	YES	YES	YES
Chadwick [41]	YES	YES	NO, but acknowledges power differentials	NO	YES	YES	NO
Daoud et al. [42]	YES	YES	NO	NO	YES	YES	NO
Dove-Medows et al. [25]	YES	NO	NO	NO	NO	YES	NO
Huschke [43]	YES	YES	NO	YES	UNDETERMINED *	YES	YES
LeMasters et al. [44]	YES	YES	NO	NO	YES	YES	NO
MacDonald et al. [45]	YES	YES	YES	NO	UNDETERMINED *	YES	NO
Mantovani & Thomas [46]	YES	YES	NO	NO	UNDETERMINED *	YES	NO
McLemore et al. [47]	YES	NO	YES	NO	YES	YES	NO
Mehra et al. [48]	YES	YES	NO	YES	YES	YES	NO
Nguyen et al. [49]	YES	YES	NO	NO	YES	YES	NO
Staneva et al. [50]	YES	YES, but only current context	YES	YES	YES	YES	YES
Stevens et al. [51]	YES	YES	NO, but discussed patient-provider power dynamics	NO	YES	NO	NO
Taylor et al. [52]	YES	YES	YES	YES	YES	YES	YES
West & Bartkowski [53]	YES	YES, but only current context	YES	YES	YES	YES	NO

* Study did not provide focus group or interview questions for us to review.

**Table 4:** Guideline Checklist to Apply Intersectionality Framework in Perinatal Health Research.

Research Stage	Questions to Consider	Approach	Example Studies in Perinatal Population	Research Resources
Conceptualization	Does study conceptualize the categories (more than one) rooted in structural and social construction?	Differences are conceptualized as primarily stemming from structural inequality (upstream) in addition to individual-level or group-level differences.	Quantitative example: Albright et al. [23] discusses how racial/ethnic minorities have a higher prevalence of chronic illness as well as more difficulty obtaining housing. Qualitative example: Macdonald et al. [45] discuss medical and social barriers to transmasculine pregnancies as the individuals transition to their preferred gender identity.	[7,8,11,54,55]
	Does the study examine the interaction of systemic forces and identities on health outcomes?	Views social categories in terms of individual/group and institutional practices rather than primarily as characteristics of individuals or groups. Examines the impact of systemic forces and identities as a unit without separating them or assuming “master” categories.	Quantitative example: Patterson et al [26] considered the historical political environment of different states within the United States when conducting their study. Qualitative example: Chadwick [41] utilized a narrative approach that examined individuals’ experiences of their intersecting identities within sociocultural discourses.	[6,11,54,55,56,57]
	Is the research topic framed within the historical and current cultural, societal, and/or situational context? ^a^	Topic background and discussion attend to social, historical, and/or global contexts of inequality.	Quantitative example: Rosenthal and Lobel [27] measured the lived experiences of gendered racism directly rather than relying on statistical analysis to account for interactions of systemic forces and identities. Qualitative example: Mehra et al. [48] described historical and current stereotypes that stigmatized Black motherhood, such as the sexist, racist presentation of the “welfare mother” and “Jezebel” (sexually aggressive).	[7,55,58]
	Does the researcher reflect on how their lived experiences and identities impact their selection of research topic and/or conceptualization?	Researcher reflects on their positionings and its impact on study conceptualization.	Qualitative example: West & Bartkowski [53] reflect on how the first author’s occupation as a doula inspired their research and how it gave insight into childbirth processes that participants discussed.	[10,14,59]
Methods	Did the researchers account for power relations in sociohistorical hierarchies, and attempt to address power differential in the decision-making process of research design and implementation?	Discuss the research design and application of intersectionality within the context of social and structural power. Includes stakeholders such as community members and/or members of the target population of study.	Quantitative example: Vedam et al. [38] incorporated a stakeholder group of community agency leaders, clinicians, and researchers to adapt a survey instrument for their study. Qualitative example: Taylor et al. [52] addressed power differences in role allocation by including a group of community members with lived experiences in the data analysis process.	[13,60,61,62]
	Does the researcher reflect on how their lived experience and identities impact the process of data collection and analysis?	Reflects on how their lived experience impact the data collection and analysis process.	Quantitative example: N/A Qualitative example: Altman et al. [24] reflected on their positions as health care providers and how it could have enriched data and analysis.	[10,13,14]
	Does the study use a method to explore the interaction of identities and systemic forces that allows for multidimensional or multilevel understanding?	Uses a method that allows multilevel and multidimensional examination. For qualitative or mixed-method approach, selects a flexible method that allows for participants to decide which identities are salient.	Quantitative example: Sen & Iyer [36] assigned a unique identity to each sub group so that it is possible to test the significance of differences between them. Qualitative example: Staneva et al. [50] encouraged participants to define their topics of concern by relating pregnancy to a “mixed bag of emotions”. They also prioritized interaction and the co-creation of the interview.	[8,10,12,13,60,63]
Interpretation/Findings	Does the researcher discuss the impact of systemic inequality on the health and mental health outcomes?	Discusses the impact of multiple systems of inequalities and how this contributes to health outcomes.	Quantitative example: Patterson et al. [26] discussed how state supportability of reproductive rights and stratified systems of health impact the health outcomes of minority populations. Qualitative example: Altman et al. [24] discussed how structural factors, such as the United States’ historically racist and patriarchal systems, impacted patient–provider communication and healthcare delivery.	[6,7,55]
	Does the researcher reflect on how their lived experiences and identities impact their interpretation/findings?	Reflects on how researchers’ identities and lived experience influence the interpretation of findings?	Quantitative example: N/A Qualitative example: Huschke [43] reflects on her experience as a doula, researcher, and activist and accounted for this in interpretation of findings by grounding them in the experiences of women from different backgrounds.	[10,14]
Dissemination (Only for researchers to think about and not necessarily for publication)	Does the research disseminate the findings to the community and/or studied and general population?	Considers a dissemination approach that increases accessibility of knowledge and makes direct impact on studied population such as discussing results at community meeting, stakeholder meeting or open-access sources.		

Note. Adapted from [7,8]. ^a^ Adapted from [20].

## Data Availability

Not applicable.
